# Phosphaturic Mesenchymal Tumor Masquerading Axial Spondyloarthritis: Diagnostic Challenge and Multidisciplinary Resolution: A Case Report

**DOI:** 10.1111/1756-185x.70783

**Published:** 2026-07-20

**Authors:** Jin Bu, Luofeng Shen, Yuhong Liu

**Affiliations:** ^1^ Department of Pediatrics, Union Hospital, Tongji Medical College Huazhong University of Science and Technology Wuhan China; ^2^ Faculty of Science, UFR Life Science University Paris Cite Paris France; ^3^ Department of Rheumatology, Union Hospital, Tongji Medical College Huazhong University of Science and Technology Wuhan China

Dear Editor,

Phosphaturic mesenchymal tumor (PMT) is a rare, insidious tumor that secretes fibroblast growth factor 23 (FGF‐23), causing chronic hypophosphatemia and tumor‐induced osteomalacia (TIO) [1, 2]. Typically small and slow‐growing, PMTs often arise in anatomically obscure locations [1], evading detection by conventional imaging and leading to delayed or misdiagnosis [3, 4]. Few cases of PMT misdiagnosed as axial spondyloarthritis (axSpA) have been reported. We herein present a 36‐year‐old Asian male initially diagnosed with axSpA for over 2 years; repeated computed tomography (CT) and magnetic resonance imaging (MRI) showed sacroiliitis, yet he failed to respond to tumor necrosis factor inhibitors (TNFi), interleukin‐17 antagonists (IL‐17Ai), and Janus kinase inhibitors (JAKi). Refractory hypophosphatemia and multiple age‐inappropriate fractures prompted re‐evaluation. Through multidisciplinary collaboration and advanced molecular imaging combined with histopathology, PMT was ultimately diagnosed and successfully resected.

The patient was admitted to our Rheumatology Department in September 2023 for an 18‐month progressive lower back, bilateral hips, and chest pain—worsened by coughing and turning at night—accompanied by morning stiffness lasting about half an hour each time and alleviating with activity. He first sought care in March 2022, visiting orthopedic, neurology, and pain clinics across multiple hospitals with no significant improvement from nonsteroidal anti‐inflammatory drugs, etc. In June 2023, the patient visited our department, where CT and MRI of the sacroiliac joints (SIJ) revealed sacroiliitis; he was diagnosed with axSpA and started on adalimumab therapy. However, symptoms persisted after 3 months, prompting admission for further evaluation. The patient had no history of chronic diseases and no family history of tumors. Physical examination showed bilateral positive Patrick signs, 3 cm chest expansion, and tenderness in both hips.

Laboratory tests showed normal erythrocyte sedimentation rate (ESR) and C‐reactive protein (CRP), serum calcium 2.03 mmol/L (normal: 2.03–2.54 mmol/L), profound hypophosphatemia (0.46 mmol/L; normal: 0.96–1.62 mmol/L), severe 25‐hydroxyvitamin D deficiency (45.68 nmol/L; normal: 75–175 nmol/L), mildly elevated parathyroid hormone (PTH), and markedly elevated alkaline phosphatase (ALP) (306 U/L; normal: 40–150 U/L). Tumor necrosis factor‐alpha (TNF‐α) (12.43 pg/mL; normal: 0.00–4.60 pg/mL) and interleukin‐6 (IL‐6) (53.00 pg/mL; normal: 0.00–5.3 pg/mL) were increased. Rheumatoid factor, anti‐citrullinated protein antibodies, antinuclear antibody, T‐SPOT.TB, and human leukocyte antigen‐B27 were negative. Dual‐energy X‐ray absorptiometry confirmed osteopenia. Chest CT showed small pericardial and pleural effusion plus bilateral cortical discontinuities in multiple ribs (Figure S1). SIJ CT showed irregular margins bilaterally (Figure 1). MRI demonstrated sacral and iliac bone edema (Figure 1) and mild bilateral hip effusion (Figure S2). Based on these findings, the patient was diagnosed with axSpA, unexplained hypophosphatemia, and possible underlying malignancy. Given no confirmed malignancy or identifiable cause of hypophosphatemia—and inadequate response to adalimumab over 3 months—treatment was switched to secukinumab, with oral phosphate and active vitamin D supplementation.

**FIGURE 1:**
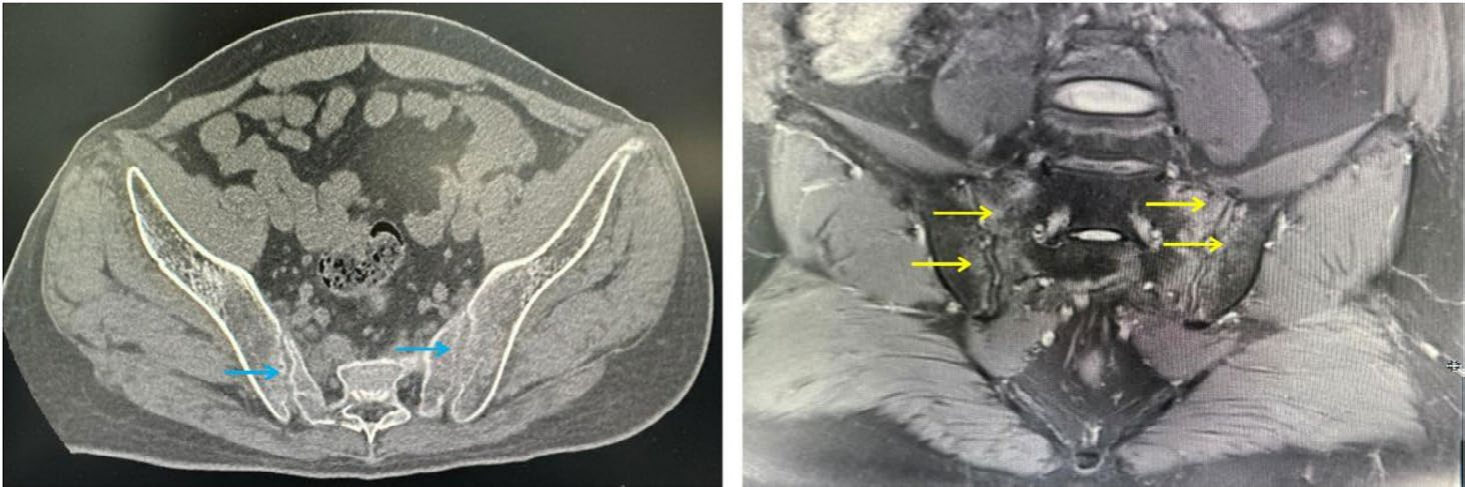
SIJ axial CT scan showed rough edges of the bilateral sacroiliac joints (blue arrow, left panel). SIJ coronal MRI scan showed bone marrow edema in the sacral and iliac regions, especially in the sacral region (yellow arrow, right panel).

After discharge, the patient had regular follow‐up at our department. Due to lack of significant symptom improvement after 3 months of secukinumab treatment, tofacitinib and upadacitinib were sequentially administered from January to June 2024, but both failed.

The patient was readmitted in July 2024 due to worsening of prior symptoms and new‐onset knee and ankle pain accompanied by muscle weakness. Physical examination showed bilateral positive Patrick sign and hip tenderness. Laboratory tests revealed normal ESR and CRP, serum calcium 2.01 mmol/L, profound hypophosphatemia (0.54 mmol/L), severe low 25‐hydroxyvitamin D deficiency (31.35 nmol/L), elevated ALP (435 U/L), mildly elevated PTH (89.1 pg/mL). Cytokine testing showed increased TNF‐α (10.65 pg/mL) and IL‐6 (21.49 pg/mL). SIJ CT showed bilateral joint surface irregularities as described above. SIJ MRI revealed axSpA‐related changes, bilateral femoral neck fractures, and possible left femoral head avascular necrosis. Based on these findings, the admission diagnoses included suspected axSpA, bilateral femoral neck fractures, left femoral head avascular necrosis, and hypophosphatemic osteomalacia of unknown cause.

Despite repeated CT and MRI findings suggestive of sacroiliitis, the patient failed to respond to multiple axSpA‐directed therapies—making axSpA unlikely. Given the patient's young age, multiple fractures, osteoporosis, low 25‐hydroxyvitamin D, and persistent hypophosphatemia. We speculate that hypophosphatemia is the primary driver of the clinical phenotype. Although vitamin D deficiency may contribute to the hypophosphatemia, preserved normocalcemia points to an atypical or rare disorder. A multidisciplinary workup (endocrinology, nuclear medicine, and orthopedic surgery) raised suspicion for TIO. The team recommended urgent surgery with concurrent biopsy for definitive diagnosis, along with scheduling molecular imaging via PET‐CT or MRI. On August 19, left hip arthroplasty was performed, but bone biopsy showed no abnormality. On August 27, ^68^Ga‐DOTATATE PET‐MRI revealed a 1.4 × 1.0 cm somatostatin receptor–positive nodule near the left femoral neck greater trochanter (SUVmax = 6.1; Figure 2). Subsequent resection in October 2024 confirmed PMT histologically (Figure 2).

**FIGURE 2:**
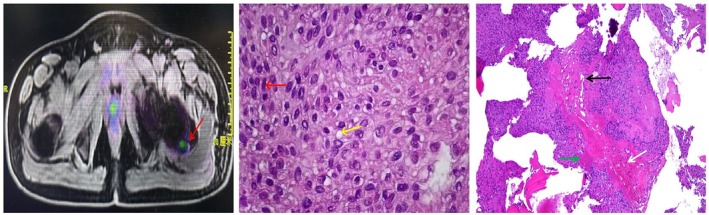
^68^Ga‐DOTATATE PET/MRI revealed an abnormal signal nodule approximately 1.4 × 1.0 cm in size near the left femoral neck greater trochanter (red arrow). The nodule showed an obvious increase in positive expression on somatostatin receptor imaging (left panel). Hematoxylin and eosin staining of the biopsied tumor specimen (original magnification ×400) showed spindle cells (red arrow) arranged in a diffuse, or fascicular pattern, alongside clusters of mature adipocytes (yellow arrow) (middle panel). At lower magnification (×100), the same specimen showed ectatic staghorn vessels (black arrow), characteristic flocculent calcifications (green arrow), and foci of metaplastic ossification (white arrow) (right panel).

Approximately 3 months postoperatively, the patient's serum phosphorus level normalized. After one‐year follow‐up, the patient's symptoms, signs, lab values (including cytokines), and imaging findings—including pericardial and pleural effusions (Figure S3), SIJ bone edema (Figure S4), and bilateral hip effusions—returned to normal, ruling out axSpA. The diagnosis was confirmed as PMT.

PMT pathogenesis centers on tumor‐driven FGF‐23 overproduction. As a phosphorus key regulatory factor, FGF‐23 disrupts phosphate homeostasis via two key mechanisms: (1) in proximal renal tubules, it binds to fibroblast growth factor receptor 1 and α‐Klotho, downregulating NaPi‐2a/NaPi‐2c and reducing renal phosphate reabsorption; (2) it suppresses 1α‐hydroxylase, lowering active vitamin D synthesis and impairing intestinal calcium and phosphate absorption [1]. These effects collectively drive TIO's biochemical and clinical features. Patients typically exhibit insidious onset and slow progression and are often misdiagnosed initially with rheumatic or neuropsychiatric disorders [5, 6]. Currently, diagnostic delays remain substantial: median time from symptom onset to definitive diagnosis is approximately 5 years, with > 95% initial misdiagnosis rates [1, 7].

Common symptoms of PMT are diffuse bone pain, progressive muscle weakness, or even fractures. PMT is rarely reported as misdiagnosed axSpA. This case's diagnostic confusion stems from three factors: (1) clinical overlap: A young man presented with low back pain, hip pain, cough‐induced chest pain, and nocturnal turning pain—symptoms highly concordant with early axSpA; (2) imaging pitfalls: CT, MRI, and PET‐MRI showed SIJ subchondral bone irregularity, bone marrow edema, and hip effusions—symptoms highly consistent with axSpA; (3) confounding biomarkers: elevated serum IL‐6 and TNF‐α, plus multiple effusions, further support a false impression of axSpA. Currently, PMT remains underrecognized by most clinicians and radiologists—largely due to its rarity, limited clinical awareness, and the complex imaging features driven by hypophosphatemia‐induced bone marrow edema, osteoporosis, cortical disruption, and even bone destruction.

The exact mechanism linking hypophosphatemia to bone marrow edema remains unclear. It is speculated that it is first related to its impact on bone metabolism, triggering local inflammation in damaged bone. Second, it impairs cellular energy metabolism—especially reduced mitochondrial ATP synthesis—leading to bone cell swelling. Whether FGF‐23 has direct proinflammatory effects requires further study. In a recent case report, Zhang et al. [8] described bilateral SIJ subchondral irregularity in PMT misdiagnosed as AS—consistent with our imaging observations—but did not specify whether SIJ edema (indicative of active inflammation) was present. Sui et al. [9], in another dedicated imaging study of sacroiliac involvement in TIO presenting with low back pain, found that all eight patients exhibited bone marrow edema concentrated in the sacral region, with bilateral symmetry and located away from the articular surface—distinct from our case. While our patient also demonstrated sacral‐predominant edema, edema extended into both sides of the SIJs. Notably, serial imaging examinations revealed concurrent pleural, pericardial, and bilateral hip joint effusions—a finding not previously reported in TIO, to the best of our knowledge, suggesting a potential pathophysiological link involving both mechanical stress arising from TIO and an inflammatory response.

Diagnosing common causes of hypophosphatemia is usually not difficult, but identifying and localizing rare causes—especially PMT—is challenging. In this case. The patient's disease progressed over 2 years despite repeated CT and MRI scans, which failed to detect a causative lesion. A bone biopsy obtained during left hip joint replacement likewise showed no tumor. PMT can lower 25‐hydroxyvitamin D, mimicking nutritional vitamin D deficiency—a finding also observed in this patient. Yet phosphate and active vitamin D supplementation failed to improve symptoms or labs. Critically, low 25‐hydroxyvitamin D alone cannot explain hypophosphatemia with normocalcemia. Additionally, prior targeted therapies ruled out axSpA. Persistent, treatment‐refractory hypophosphatemia prompted advanced diagnostic evaluation. Through a multidisciplinary consultation and somatostatin receptor‐targeted molecular imaging (^68^Ga‐DOTATATE PET‐MRI), the tumor was localized to the left femoral greater trochanter with high sensitivity. Surgical re‐biopsy confirmed the diagnosis of PMT. Post‐resection follow‐up demonstrated rapid and sustained normalization of the patient's laboratory parameters, imaging findings, and clinical symptoms.

Due to rarity, nonspecific symptoms, and occult lesions, PMTs are often misdiagnosed or mistreated as rheumatic diseases for years. This case highlights three key points: (1) PMT should be suspected in patients presenting with unexplained hypophosphatemia, normocalcemia, elevated alkaline phosphatase, and symptoms such as musculoskeletal pain, severe limb weakness, or age‐inappropriate fractures. (2) PMT can arise in obscure locations. Early diagnosis requires heightened clinical vigilance, timely assessment of phosphate metabolism and serum FGF‐23, and targeted imaging—especially ^68^Ga‐DOTATATE PET‐MRI/CT—with multidisciplinary collaboration essential to avoid misdiagnosis. (3) Typical axSpA symptoms and SIJ changes do not necessarily indicate axSpA. If treatment fails, re‐evaluate for alternative diagnoses.

## Author Contributions

Jin Bu: writing – original draft, writing – review and editing. Luofeng Shen: writing‐original draft. Yuhong Liu: resources, supervision, writing – review and editing.

## Funding

The work was supported by the National Natural Science Foundation of China (NSFC) (grant no. 82301782); Natural Science Foundation of Hubei Province (grant no. 2023AFB494).

## Ethics Statement

The article is a case report; therefore, approval from the Ethics Review Board at Union Hospital, Tongji Medical College, Huazhong University of Science and Technology was waived.

## Consent

A written patient consent was obtained from the patient for publication of this case report and any accompanying images.

## Conflicts of Interest

The authors declare no conflicts of interest.

## Supporting information


**Figure S1:** CT scan showed a small amount of pericardial effusions (white arrow) (left panel) and pleural effusion on both sides (yellow arrow) (left panel) plus cortical discontinuities in multiple ribs (white arrow) (right panel).
**Figure S2:** CT scan showed a small amount of bilaterial hip effusion (yellow arrow).
**Figure S3:** Chest CT at one‐year follow‐up post‐surgery demonstrates bilateral pleural thickening, with complete resolution of both pericardial and pleural effusions.
**Figure S4:** SIJ CT performed at the one‐year postoperative follow‐up revealed no significant structural abnormalities (Metal artifacts can be observed in the left iliac bone area, red arrow) and complete resolution of previously documented sacral and iliac bone marrow edema.

## Data Availability

The data that support the findings of this study are available on request from the corresponding author. The data are not publicly available due to privacy or ethical restrictions.
