# Supplementary material for: Phosphaturic Mesenchymal Tumor Masquerading Axial Spondyloarthritis: Diagnostic Challenge and Multidisciplinary Resolution: A Case Report
Source: Int J Rheum Dis. 2026 Jul 20;29(7):e70783. doi: 10.1111/1756-185x.70783 (PMC13382479; doi:10.1111/1756-185x.70783)
Supplement: Supplementary file 1 — Figure S1: CT scan showed a small amount of pericardial effusions (white arrow) (left panel) and pleural effusion on both sides (yellow arrow) (left panel) plus cortical discontinuities in multiple ribs (white arrow) (right panel). Figure S2: CT scan showed a small amount of bilaterial hip effusion (yellow arrow). Figure S3: Chest CT at one‐year follow‐up post‐surgery demonstrates bilateral pleural thickening, with complete resolution of both pericardial and pleural effusions. Figure S4: SIJ CT performed at the one‐year postoperative follow‐up revealed no significant structural abnormalities (Metal artifacts can be observed in the left iliac bone area, red arrow) and complete resolution of previously documented sacral and iliac bone marrow edema. [file APL-29-e70783-s001.docx]

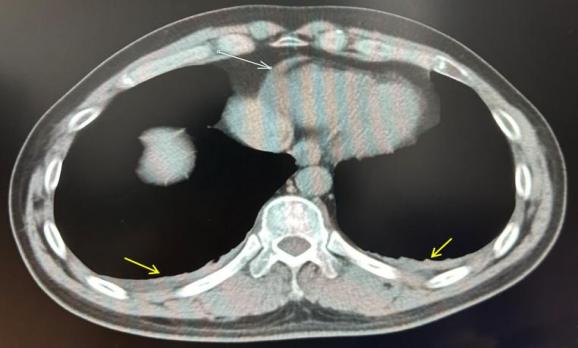

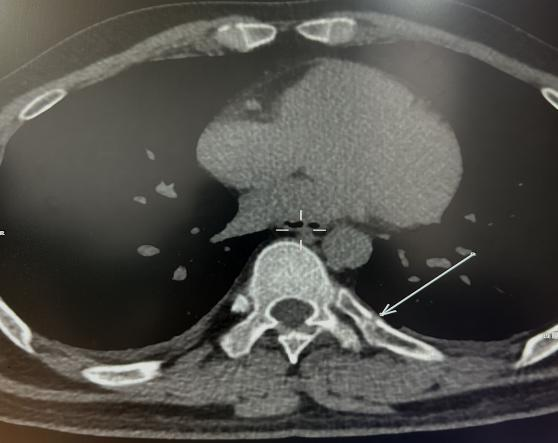


FIGURE S1 CT scan showed a small amount of pericardial effusions (white arrow) (left panel) and pleural effusion on both sides (yellow arrow) (left panel) plus cortical discontinuities in multiple ribs (white arrow) (right panel).


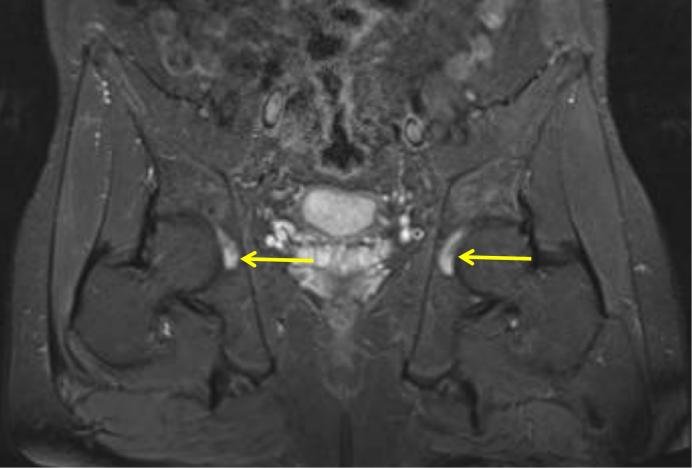


FIGURE S2 CT scan showed a small amount of bilaterial hip effusion (yellow arrow)


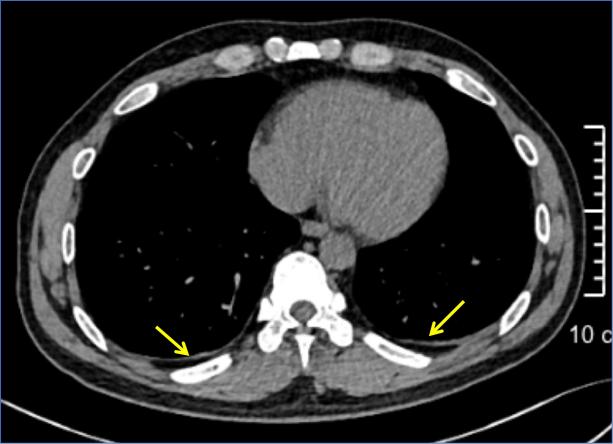


FIGURE S3 Chest CT at one-year follow-up post-surgery demonstrates bilateral pleural thickening, with complete resolution of both pericardial and pleural effusions.


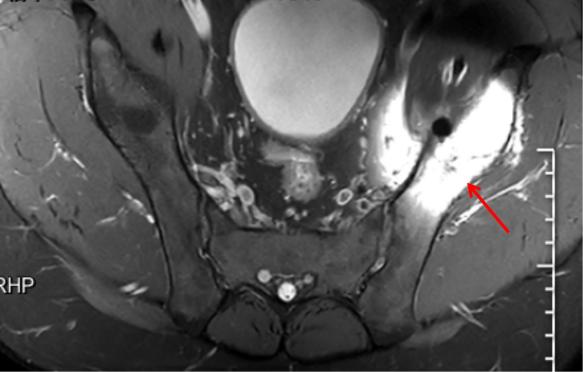

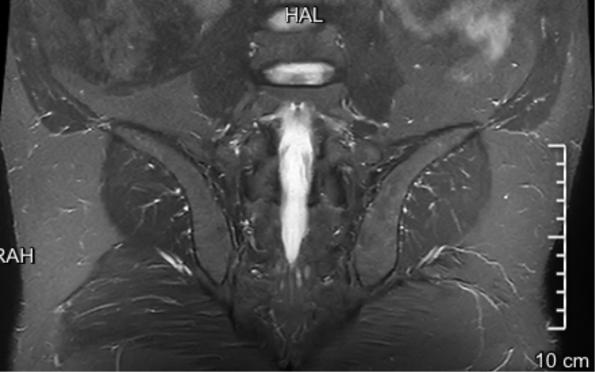


FIGURE S4 SIJ CT performed at the one-year postoperative follow-up revealed no significant structural abnormalities (Metal artifacts can be observed in the left iliac bone area, red arrow) and complete resolution of previously documented sacral and iliac bone marrow edema.
